# Optimization and Preliminary Physicochemical Characterization of Pectin Extraction from Watermelon Rind (*Citrullus lanatus*) with Citric Acid

**DOI:** 10.1155/2022/3068829

**Published:** 2022-01-06

**Authors:** José Pérez, Karina Gómez, Lorena Vega

**Affiliations:** Department of Chemical Engineering, Bioprocess Research Group, Universidad del Atlántico, Puerto Colombia 081001, Colombia

## Abstract

Watermelon rind was used for the pectin extraction with citric acid as the extractant solvent. The effects of pH (2.0-3.0), extraction time (45-75 min), and liquid-solid ratio (10 : 1 to 40 : 1 mL/g) on the pectin yield, degree of esterification, methoxyl content, and anhydrouronic acid content were investigated using Box-Behnken surface response experimental design. The pH was the most significant variable for the pectin yield and properties. The responses optimized separately showed different optimal conditions for each one of the variables studied in this work. Therefore, the desirability function was used to determine the sole theoretical optimum for the highest pectin yield and highest anhydrouronic acid content, which was found to be pH of 2.0, extraction time of 62.31 min, and liquid-solid ratio of 35.07 mL/g. Under this optimal condition, the pectin yield, degree of esterification, methoxyl content, and anhydrouronic acid content were 24.30%, 73.30%, 10.45%, and 81.33%, respectively. At optimal conditions, watermelon rind pectin can be classified as high methoxyl and rapid-set pectin with high quality and high purity. *Practical Applications*. This study evaluated the pectin extraction from watermelon rind and carried out an optimization of multiple responses as a function of pH, time, and liquid-solid ratio to obtain the best preliminary quality parameters (pectin yield and anhydrouronic acid content). The results revealed that watermelon rind waste can be an inexpensive source to obtain good pectin quality and high purity. According to the chemical characterization and physicochemical properties studied, the extracted pectin from watermelon rind would have a high potential to be used in food industry.

## 1. Introduction

Watermelon (*Citrullus lanatus*) is a Cucurbitaceae, creeping herbal plant or climbing plant characterized as a large and juicy fruit. Watermelon fruit is composed of flesh (edible part), seeds distributed throughout the flesh, and the rind representing 30-40% of the total weight [1, 2]⁠. Health benefits such as prevention against cardiovascular diseases are attributed to the fruit [3, 4]⁠. The literature has determined that the watermelon seeds [5]⁠ and watermelon rinds [6]⁠ contain antioxidant properties. Watermelon is mainly used for local production of juices, which generate large amounts of waste without a proper disposal treatment. These watermelon residues have great potential to produce pectin and other value-added products.

Pectin is a linear polysaccharide mainly composed of anhydrogalacturonic acid units with *α*(1⟶4) bonds, and the carboxyl acid groups are esterified with methyl groups partially [7]. Its structure includes other components such as neutral sugars: arabinose, galactose, rhamnose, and xylose [7, 8]⁠, which can react with methanol to form methyl esters or neutralized by a base [9]⁠. Pectin has gelling properties that are used for the aqueous gel formation in the food industry [10, 11]⁠. Some of the pharmaceutical applications of pectin are to diminish lipid digestion [12]⁠, to improve lipid hepatic accumulation [13]⁠, glucose tolerance for antidiabetic effects [14]⁠, and its anti-inflammatory activity in high methoxyl pectins [15]⁠. Recently, the low hydrophilic of pectin has taken advantage to improve carrier properties and diffusion control of nicotine in transdermal patches [16, 17]⁠ and drug delivery such as theophylline tablets [18]⁠ and lidocaine and aspirin in ionic liquid [19]⁠.

The variables that affect the extraction yield are pH, temperature, time, and liquid-solid ratio (LSR). The quality parameters of pectin include extraction yield and properties such as ash content [20]⁠, free acidity [21]⁠, methoxyl content [22]⁠, degree of esterification [23]⁠, anhydrogalacturonic acid content [24, 25],⁠ and Fourier transform infrared spectroscopy (FTIR) analysis [26, 27]⁠. Pectin is classified through its degree of esterification (DE) due to define its gelling properties. High methoxyl pectins are characterized by forming gels in high soluble solids and acidic systems, which have galacturonic acid units that are more than 50% (DE > 50%) esterified with methyl groups. In low methoxyl pectins (DE < 50%), the gelation occurs on a widespread pH range than high methoxyl pectins and requires the presence of divalent cations [7, 8, 28]⁠.

The primary sources of industrial pectin extraction are apple pomace and citrus peels [28, 29]⁠. However, pectin from agroindustrial process wastes is generating a great interest because their use can reduce environmental impacts provoked by themselves and give added value to the agroindustrial production chain. Many biological residues such as pomelo peels [30, 31]⁠, banana peels [32]⁠, mango peels [33, 34]⁠, melon peels [11]⁠, durian fruit-hulls [35],⁠ and heads of sunflower [25]⁠ have been utilized to obtain pectin. Hence, watermelon rind can be used to produce pectin, while adding value to the watermelon's agribusiness.

Several works have reported the extraction of pectin from watermelon wastes by using different methods such as acid hydrolysis [36, 37] and assisted microwave extraction [38, 39]⁠. However, the acid hydrolysis is the most employed method for the pectin extraction from food waste. Therefore, the objectives of this study are to optimize and characterize the pectin extraction from watermelon rind using citric acid as solvent for extraction through the response surface methodology (RSM) and determine optimal conditions for the highest pectin yield and anhydrouronic acid content (AUA) simultaneously.

## 2. Materials and Methods

### 2.1. Sample Preparation

Watermelon rinds were obtained from a local market of watermelon juices located in the Barranquilla center and around the Universidad del Atlántico, Colombia. They were washed, manually cut, and heated in distilled water until boiling to denature enzymes and inactive microorganisms [40]⁠. The collected material was milled for homogenization and was dried in a convection oven at a temperature no higher than 80°C for 24 hours. Finally, the treated watermelon rind was packed and stored in a desiccator for later use.

### 2.2. Chemicals and Solvents

All chemicals and solvents such as citric acid, ethanol, and methanol used were of analytical grade.

### 2.3. Pectin Extraction

The pectin extraction was carried out through acid hydrolysis with citric acid, according to the methodology with some modifications [41]⁠. The dried watermelon rind was stirred in citric acid solutions with the following defined conditions for all runs of the Box-Behnken design: pH (2.0, 2.5, and 3.0); extraction time (45, 60, and 75 min), and liquid-solid ratio (10 : 1, 25 : 1, and 40 : 1 mL/g) at a constant temperature of 80°C. The resultant slurry was vacuum filtered with a microcloth using vacuum pressure. The residual liquid was precipitated with methanol in a 60% volume solution. The obtained precipitate was washed three times with 70% ethanol and was subsequently rewashed with 96% ethanol. The collected precipitate was dried in a convection oven until constant weight at 50°C for 12 hours.

### 2.4. Pectin Yield

The pectin yield of watermelon rind on a dry weight basis was determined as follows [10, 42]⁠:

(1) $$\begin{array}{c} \text{Pectin yield}=\frac{\text{weight of dried pectin }\left(\text{g}\right)}{\text{weight of dried watermelon ring }\left(\text{g}\right)}\times 100, \end{array}$$

where the dried pectin was obtained after the treatment of filtration, precipitation, and drying; the dried watermelon rind was collected as result of sample preparation.

### 2.5. Pectin Characterization

#### 2.5.1. Determination of Degree of Esterification and Anhydrouronic Acid Content

The degree of esterification and the anhydrouronic acid content were determined by titration relating the methoxyl content with the equivalent weight. 0.50 g of watermelon rind pectin was dissolved into ethanol/water solution (1 : 20 *v*/*v*); 5 drops of phenol red indicator were added, and the sample was titrated with 0.1 N sodium hydroxide (*V*_1_, mL) until the indicator changed. Then, 25 mL of 0.25 N NaOH was added, and the sample was heated and stirred vigorously. Five drops of phenol red and 25 mL of 0.25 N HCl were added again, and it was titrated with 0.1 N NaOH (*V*_2_, mL) until the color changes from yellow to faint pink endpoint [43]⁠. The MeO and the DE were then calculated according to Equation (2) [22, 31]⁠ and Equation (3) [11, 23, 44]⁠, respectively.

(2) $$\begin{array}{c} \text{MeO }\left(\%\right)=\frac{V_{2}\left(\text{mL}\right)0.1\left(\text{N}\right)31}{\text{weight of sample }\left(\text{mg}\right)}\times 100, \end{array}$$

(3) $$\begin{array}{c} \text{DE }\left(\%\right)=\frac{V_{2}\left(\text{mL}\right)}{V_{1}\left(\text{mL}\right)+V_{2}\left(\text{mL}\right)}\times 100. \end{array}$$

Equation (4) was used to calculate the anhydrouronic acid content [22]⁠.

(4) $$\begin{array}{c} \text{AUA }\left(\%\right)=\frac{0.1\left(\text{N}\right)\left(V_{1}\left(\text{mL}\right)+V_{2}\left(\text{mL}\right)\right)176}{\text{weight of sample }\left(\text{mg}\right)}\times 100, \end{array}$$

where 176 is the molecular weight of anhydrouronic acid expressed as mg/meq and *V*_1_ and *V*_2_ were the volumes used for first and second titration, respectively.

### 2.6. Fourier Transform Infrared Spectroscopy

Fourier transform infrared (FTIR) spectra of the extracted pectin were used to evaluate its structural chemical properties. FTIR analysis was performed in the SHIMADZU (IRAffinity-1) spectrometer with a resolution of 4 cm^−1^ and 130 scans of wavelengths ranging from 4000 to 400 cm^−1^.

### 2.7. Experimental Design

The Box-Behnken design response surface design was used to evaluate the effect of three independent variables such as pH (*X*_1_: 2.0-3.0), the extraction time (*X*_2_: 45-75 min), and the liquid-solid ratio, LSR (*X*_3_: 10-40 mL/g) on simultaneous responses (pectin yield, the MeO, the DE, and AUA).  Table 1 summarizes the levels and code of three independent variables.

The experimental design and variance analysis (ANOVA) were performed using R-programming software 4.0.2. The response variables were fitted to the second-order polynomial model as given for the following Equation (9):

(5) $$\begin{array}{c} Y=B_{0}+\overset{3}{\underset{i=1}{\sum }}B_{i}X_{i}+\overset{3}{\underset{i=1}{\sum }}B_{ii}X_{i}^{2}+\overset{2}{\underset{i=1}{\sum }}\overset{3}{\underset{j=i+1}{\sum }}B_{ij}X_{i}X_{j}, \end{array}$$

where *Y* is the response variable; *B*_0_, *B*_*i*_, *B*_*ii*_, and *B*_*ij*_ are the coefficient terms for the regression model; and *X*_*i*_ and *X*_*j*_ are the levels of independent variables. The responses were initially optimized separately to predict the 3D surface and the maximum values. Latterly, an optimization of simultaneous responses using the desirability function [45]⁠ was performed.

## 3. Results and Discussion

### 3.1. Experimental Results and Quality Parameters

Table 2 presents the experimental values obtained for pectin yield, methoxyl content, degree of esterification, and anhydrouronic acid content at each Box-Behnken design (BBD) point. The pectin yield range (4.19-27.86%) was within the typical values reported of 15-20% and 30-35% for dried apple pomace and citric peels, respectively [28]⁠. The results indicate that the watermelon rind pectin can be classified as high methoxyl pectin [29]⁠ due to its DE and methoxyl content (MeO) which were higher than the reference values of 50% and 6.7% [46]⁠, respectively. Furthermore, according to the Food Agriculture Organization (FAO) and the World Health Organization (WHO), the AUA suggests that a high-quality pectin (AUA > 65% FAO/WHO) was obtained [47]⁠, except for runs carried out at pH 3.0.

### 3.2. Statistical Analysis

The analysis of variance (ANOVA) of the results was used to determine the effects of pH (*X*_1_), extraction time (*X*_2_), and liquid-solid ratio (*X*_3_) on each of the responses of watermelon rind pectin (Table 3). ANOVA revealed that the linear effect (*X*_1_) was significant (*p* < 0.05) for pectin yield, MeO, and AUA. Interactions (*X*_2_ : *X*_3_) had significant for DE and MeO. Furthermore, square effects *X*_1_^2^ and *X*_2_^2^, *X*_1_^2^ and *X*_3_^2^, and*X*_2_^2^ were also significant for MeO, AUA, and DE, respectively.

By using multiple regression analysis on the experimental responses, the second-order model was utilized to fit experimental data and predict the effects of dependent variables. The validity of the model could be confirmed because the lack-of-fit tests were not significant, and the following determination coefficients (*R*^2^) 0.9162, 0.8841, 0.9338, and 0.9301 were obtained for pectin yield, DE, MeO, and AUA, respectively. These results show that the calculated responses by the model were reliable and adequate. The second-order equations (coded factors) for pectin yield (Equation (6)), DE (Equation (7)), MeO (Equation (8)), and AUA (Equation (9)) were given as follows

(6) $$\begin{array}{c} \text{Pectin yield}\left(\%\right)=11.99-7.32X_{1}+0.65X_{2}+1.17X_{3}-0.89X_{1}X_{2}-2.69X_{1}X_{3}-0.92X_{2}X_{3}+3.41X_{1}^{2}-3.03X_{2}^{2}-2.42X_{3}^{2}, \end{array}$$

(7) $$\begin{array}{c} \text{DE}\left(\%\right)=73.67-0.17X_{1}+0.025X_{2}+1.47X_{3}+0.82X_{1}X_{2}+0.50X_{1}X_{3}+3.99X_{2}X_{3}-2.48X_{1}^{2}-3.10X_{2}^{2}+1.74X_{3}^{2}, \end{array}$$

(8) $$\begin{array}{c} \text{MeO}\left(\%\right)=10.33-1.26X_{1}-0.16X_{2}+0.28X_{3}+0.21X_{1}X_{2}-0.22X_{1}X_{3}+0.99X_{2}X_{3}-1.24X_{1}^{2}-0.97X_{2}^{2}-0.57X_{3}^{2}, \end{array}$$

(9) $$\begin{array}{c} \text{AUA}\left(\%\right)=79.59-9.87X_{1}-1.40X_{2}+0.80X_{3}+0.78X_{1}X_{2}-1.93X_{1}X_{3}+3.96X_{2}X_{3}-7.24X_{1}^{2}-4.81X_{2}^{2}-5.97X_{3}^{2}. \end{array}$$

### 3.3. Effect of Independent Variables on Pectin Yield

**Figure 1:**
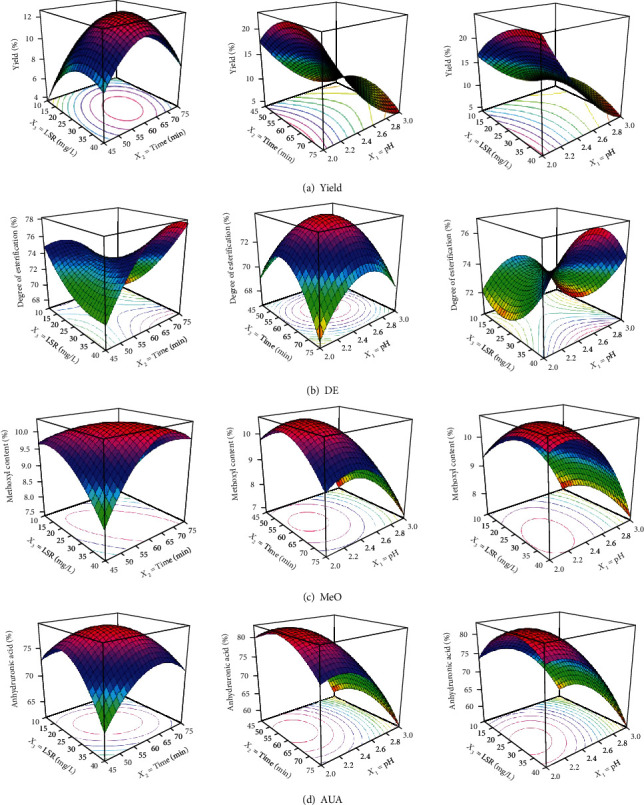
Response surface showing the effect of dependent variables (pH, time extraction, and LSR) on the pectin yield (a), DE (b), MeO (c), and AUA (d).

ANOVA showed that pH (linear effect) was the only variable that affected pectin yield significantly [48]⁠. The determination coefficient indicates that 91.62% of the total variation can be explained by the quadratic model (Equation (6)), which suggests that the model is suitable to predict the yield pectin from watermelon rind under the experimental conditions evaluated in this work.  Figure 1(a) shows that the pectin yield was favored at lower pH (negative regression term), and the higher pectin yield was obtained at the lowest pH of 2.0. The carboxyl groups present in pectin are hydrated due to the acidified extraction solvent at lower pH; the loss of charges in carboxyl groups tends to reduce the repulsive forces, promoting the pectin precipitation [10]⁠. Indeed, more pectin dissolution by hydrolysis of nonsoluble pectin and increasing of pectin mass transfer from the plant source at lowering pH are shown by the literature [23, 24, 30, 48, 49]⁠. These conditions were shown to have led to increase the pectin release and recovery.

These results were similar to other reported pectins, which showed a significant rise of pectin yield at lower pH for extracted pectin from mango peels [33]⁠ and watermelon rind [38]⁠.  Figure 1(a) illustrates that at high points of extraction time (*X*_2_) and low values of LSR (*X*_3_), the pectin yield decreased; this behavior is in agreement with results reported in the pectin extraction from seed watermelon peel [50]⁠ and pectin extraction from carrot pomace [23]⁠. This is likely because of extended extraction time; the citric acid causes the breakdown of glycoside bonds and ester bonds of pectin [51]⁠. On the other hand, excessive or insufficient amounts of LSR influenced the pectin mass transfer due to the solution saturation or low dissolution capacity. Therefore, the pectin yields were smaller at extreme LSR conditions [50].

### 3.4. Effect of Independent Variables on DE

As can be seen, in Table 2 and Figure 1(b), the extracted pectin is considered like a high methoxyl pectin with DE ranging from 66.67% to 75.44%. According to ANOVA, linear effects were not significant, and the interaction between time extraction and LSR (*X*_2_ : *X*_3_) and the quadratic effect of the time extraction (*X*_2_^2^) affected the DE (Table 3(a)). High methoxyl pectin (DE > 50%) has been obtained from pomelo peel [30]⁠, pomelo albedo [21]⁠, and watermelon rind [36], indicating that our findings are in agreement with these previous works. It was noticed that the DE increased for more extensive extraction time. These results are in accordance with the reported values given in pectin from cocoa husks [20]⁠. Under the conditions tested in this study, the DE showed that the obtained watermelon rind pectin was characterized with a low degree of deesterification of polygalacturonic chains.

### 3.5. Effect of Independent Variables on MeO

The methoxyl content listed in Table 2 varied from 6.75 to 10.66%. Based on the regression analysis (Equation (8)), it can be suggested that pH (linear and quadratic coefficients) negatively influenced the MeO (Figure 1(c)). In other words, decreasing pH led to get higher the methoxyl content. Other significant factors were interaction coefficient terms (*X*_2_ : *X*_3_, Figure 1(c)) and quadratic coefficient of the time extraction (*X*_2_^2^). In addition, the *R* squared pointed out that 6.62% of data variability could not be explained by Equation (8). The acidic extraction (citric acid) was favorable towards the methoxylation of side acid groups of polygalacturonic chains. This is probably due to the methoxylation which was catalyzed by acidity medium (low pH), which increases the reactivity of the acid groups by shifting the equilibrium forward to methyl ester formation. The methoxyl content of citrus peel pectin and apple pectin [52]⁠ was comparable with obtained results.

### 3.6. Effect of Independent Variables on AUA

Similar to the pectin yield, pH was the most significant variable for the anhydrouronic acid content. Unlike the yield, the quadratic coefficient term of LSR (*X*_3_^2^) was also significant. In Figure 1(d), it was observed that AUA (purity pectin) increased at lower levels of pH and low-intermediate levels of LSR. However, the highest value of AUA (82.50%) was obtained at the lowest pH (2.0), greatest LSR (40 mL/g), and time extraction of 60 minutes. Several works from different plant sources like orange peels [49]⁠, ponkan peels [24]⁠, and banana peels [32]⁠ have shown that low pH and LSR between 20 and 25 mL/g increased the AUA [20, 24, 49]. By contrast, it has been previously shown that longer extraction time improved pectin purity [20, 25, 32]⁠, which did not agree with the results in the present study.

### 3.7. Pectin Optimization

Based on the polynomial models fitted for each of the responses, a separate optimization process was carried out to find out the optimal conditions that maximized pectin yield, DE, MeO, and AUA independently. Different optimal conditions were obtained (Table 4 and Figure 1). These optimal predictions confirmed the strong influence of pH (low values) on the pectin yield, MeO content, and AUA content as well as previously shown. It is noteworthy that for all responses except DE, the optimal conditions were at low pH, intermediate values of time extraction (nearly 60 min), and midpoints to high levels of LSR for the study regions tested in this work.

In order to unify to one optimal condition (theoretical optimum), an analysis of the multiple responses through the desirability discontinuous functions [45]⁠ using R-programming software [53] was performed. The conditions obtained for the highest pectin yield and highest AUA simultaneously were found at pH of 2.0, extraction time of 62.31 min and LSR of 35.07 g/mL (Table 4). Theoretical optimum was quite similar to the optimal conditions predicted for pectin yield model in Equation (6) as is shown in Table 4. In fact, the theoretical optimum pectin yield (24.30%) can be considered equivalent to highest predicted by Equation (6), which is slightly lower than the experimental value, 27.16%. In addition, regarding the other responses, the theoretical predictions are very good, similar to the experimental values (Table 2). According to these findings, the models were well fitted to predict the effect of independent variables and their responses.

The optimal pectin yield (24.30%) was comparable with other studies that also used the desirability function to optimize simultaneous responses. The pectin extraction from melon peels [11]⁠, mango peels [34],⁠ and ponkan peels [24]⁠ reported maximum pectin yields of 29.48%, 30.0%, and 25.6%, respectively. Previous works from watermelon rind have reported pectin yields of approximately 19% [38]⁠ and 25% [37, 39]⁠. It has shown that citric acid was a better solvent extractant than hydrochloric acid in the pectin extraction from watermelon rind [36]⁠. Nevertheless, their maximum pectin yield (8.38%) [36]⁠ obtained was very low compared to the reported values by this study and previous works, despite their optimal conditions (pH of 2.0, extraction time of 180 min, LSR of 25, and temperature of 80°C) [36]⁠, which were pretty similar to ours. This might be due to the Colombian variety of watermelon and using methanol as a precipitating agent, which has shown to be a better precipitating agent than ethanol in the extraction of pectin from pomelo albedo [21]⁠. For this reason, the highest properties of pectin were obtained from watermelon rind.

### 3.8. Characterization of Pectin

The characteristics of extracted pectin from watermelon rind using citric acid under the highest yield experimental conditions (pH of 2.0, time of 60 min, and LSR of 40 mL/g) were comparable with commercial pectin (Merck).  Table 5 shows the remarkable properties of the pectin obtained. The ash content and alkalinity of ash were 1.24% and 0.39%, respectively, which were lower than the accepted levels for the standard pectin. The ash content is a measure of the degree of purity [52]⁠ and quality [42]⁠ of pectin. Based on these results, the extracted pectin presented low amounts of soluble solids and high purity. Free acid registered a high value compared to standard pectin; this might be due to the chemical nature of carboxyl groups, which in harsh pH conditions are hydrolyzed, increasing pectin acidity.

High methoxyl and rapid-set pectin was obtained from watermelon rind because of the MeO (10.66%), and DE (73.33%) values are ranged for this pectin type (Table 5). Rapid-set pectin is used in high-sugar jams, jellies, and marmalades [7, 8]⁠, indicating that pectin from watermelon rind could likely be employed in food applications. High methoxyl pectin from watermelon rind has been previously reported [36–39]⁠, and these findings are in agreement with obtained results. From the results of AUA, the extracted pectin had a value of 82.5%, which was higher than those reported by similar works with watermelon rind [36–38]⁠ and as well as the percent requirements for commercial food or pharmaceutical purpose [46, 47]⁠. It should be pointed out that the AUA is a determining quality parameter and gelling properties of pectin [23, 46]⁠. Therefore, the results related to AUA also indicated that high-quality pectin was extracted from watermelon rind by using citric acid.

### 3.9. FTIR Analysis

**Figure 2:**
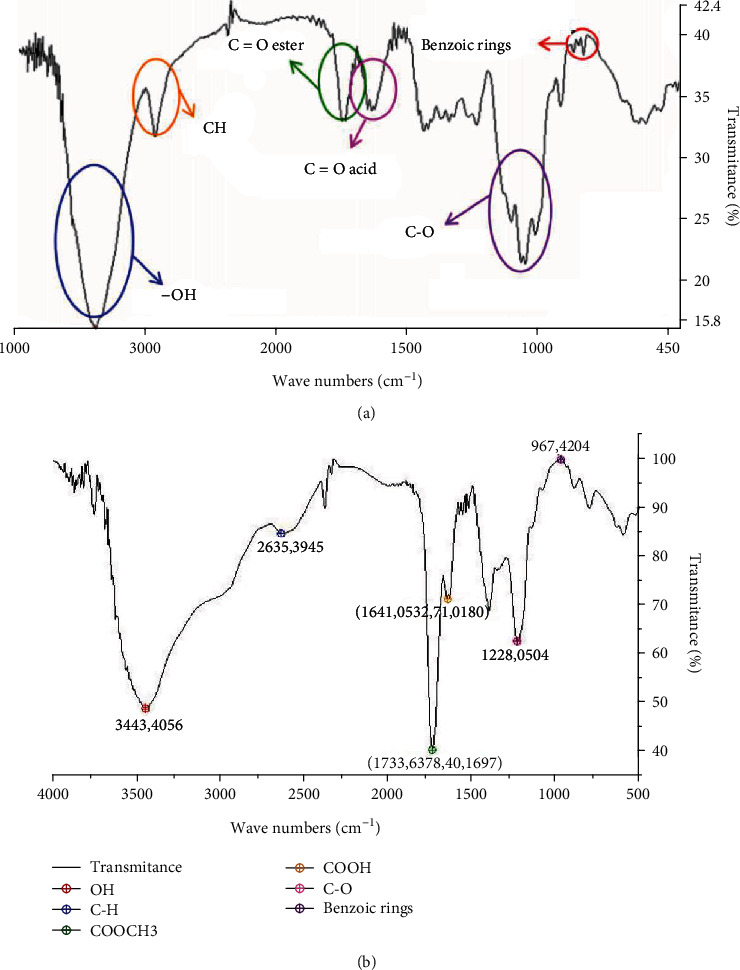
FTIR spectra of pectin: (a) commercial rapid-set pectin and (b) extracted pectin from watermelon rind.

According to the properties shown for the extracted pectin in this work (Table 4), the FTIR spectra were compared to commercial rapid-set pectin [54]⁠ as is illustrated in Figure 2. FTIR analysis at highest yield experimental conditions (pH of 2.0, time extraction of 60 min, and LSR of 40 mL/g) identified the main functional groups present in this type of pectin. The peaks related to O-H and C-H stretching vibrations were between 3500-3250 cm^−1^ and 3000-2700 cm^−1^, respectively. Stretching vibrations (=CO) of esterified carboxyl groups and free carboxyl groups (1800-1600 cm^−1^) were observed in the fingerprint regions of the pectin spectrum [10, 55–57]⁠. The peak of CH bending vibrations for pyranose ring (approximately 1338 cm^−1^) and the peak of COO^−^ stretching vibration for ester groups (1330-1210 cm^−1^) can be assigned in Figure 2 [55]⁠. Furthermore, the characteristic overlapped peaks of glycoside bonds and pyranose cycles around 1000 cm^−1^ and weak peaks (830-500 cm^−1^) associated with *α*- and *β*-configurations were identified, indicating that the pectin is the principal component [55, 58]⁠. The bands of FTIR spectra of the obtained pectin were similar to reported with analogous work of pectin extraction from watermelon rind [36].

## 4. Conclusions

In this study, the pectin extracted from watermelon rind with citric acid is considered a high methoxyl pectin (DE > 50% and MeO > 6.7%) and has high quality (AUA > 65%, FAO/WHO, except for runs at pH of 3.0). Based on the results of BBD, pH was the most significant variable on the pectin yield and its properties. The simultaneous optimization to obtain the highest pectin yield and highest AUA showed that the optimal conditions were found at pH of 2.0 (lowest level), extraction time of 62.31 min (intermediate points), and 35.07 mL/g (high- midpoints), which were close to optimal experimental conditions (pH of 2.0, time of 60 min, and LSR of 40 mL/g) for the highest yield pectin. Under this optimum pectin yield, DE, MeO, and AUA were 24.32%, 73.33% (rapid-set pectin), 10.66, and 82.50% (AUA > 74% USP, high quality), respectively. In addition, ash content and alkalinity of ash revealed high purity of pectin at the optimal condition.

## Figures and Tables

**Table 1:** Levels and code of variables chosen for the Box-Behnken design.

Dependent variables	Real values of coded levels		
	-1	0	1
*X*_1_: pH	2.0	2.5	3.0
*X*_2_: time (min)	45	60	75
*X*_3_: liquid-solid ratio, LSR (mL/g)	10 : 1	25 : 1	40 : 1

**Table 2:** Experimental conditions of pectin extraction with citric acid and responses for the Box-Behnken experimental design.

Run	Independent variables			Experimental responses			
	*X*_1_^a^	*X*_2_^b^ (min)	*X*_3_^c^ (g/mL)	Yield (%)	DE (%)	MeO (%)	AUA (%)
1	2.0	45	25	17.02	71.13	9.91	79.11
2	3.0	45	25	5.31	67.71	7.45	62.49
3	2.0	75	25	21.23	66.83	8.36	71.02
4	3.0	75	25	5.95	66.67	6.75	57.52
5	2.0	60	10	14.59	71.42	9.39	74.68
6	3.0	60	10	4.19	71.53	6.82	54.11
7	2.0	60	40	27.16	73.33	10.66	82.50
8	3.0	60	40	5.99	75.44	7.21	54.23
9	2.5	45	10	6.77	73.43	9.40	72.69
10	2.5	75	10	8.80	68.22	7.89	65.69
11	2.5	45	40	6.12	68.42	7.71	64.00
12	2.5	75	40	4.47	79.17	10.16	72.83
13	2.5	60	25	11.48	73.47	10.51	81.24
14	2.5	60	25	11.50	74.61	10.44	79.46
15	2.5	60	25	13.00	72.93	10.03	78.08

^a^ *X*_1_ (pH with three levels: 2.0, 2.5, and 3.0), ^b^*X*_2_ (extraction time with three levels: 45, 60, and 75 minutes), and ^c^*X*_3_ (LSR with three levels: 10, 25, and 40 mL/g).

**(a):** 

Source	Pectin yield (%)					DE (%)				
	Df	Sum sq	Mean sq	*F* value	Pr (>*F*)	Df	Sum sq	Mean sq	*F* value	Pr (>*F*)
Model	9	584.37	64.93	6.08	0.0306^∗^	9	156.22	17.36	4.24	0.0631
Linear	3	443.10	147.70	13.82	0.0074^∗∗^	3	17.52	5.84	1.43	0.3911
Interaction	3	35.57	11.86	1.11	0.4274	3	67.34	22.45	5.48	0.0488^∗^
Square	3	105.70	35.23	3.30	0.1159	3	71.36	23.79	5.81	0.0438^∗^
Residuals	5	53.44	10.69			5	20.48	4.10		
Lack of fit	3	51.92	17.31	22.77	0.0424	3	19.00	6.33	8.61	0.1058
Pure error	2	1.52	0.76			2	1.47	0.74		
Total	28	1275.62				28	353.39			

**(b):** 

	MeO (%)					AUA (%)				
	Df	Sum sq	Mean sq	*F* value	Pr (>*F*)	Df	Sum sq	Mean sq	*F* value	Pr (>*F*)
Model	9	27.00	3.00	7.40	0.0201^∗^	9	1238.65	137.63	7.84	0.0177^∗^
Linear	3	13.57	4.52	11.15	0.0118^∗^	3	800.20	266.73	15.19	0.0060^∗∗^
Interaction	3	4.29	1.43	3.53	0.1041	3	79.90	26.63	1.52	0.3184
Square	3	9.14	3.05	7.51	0.0267^∗^	3	358.55	119.52	6.81	0.0323^∗^
Residuals	5	2.03	0.41			5	87.80	17.56		
Lack of fit	3	1.89	0.63	9.39	0.0978	3	82.78	27.59	10.99	0.0845
Pure error	2	0.13	0.07			2	5.02	2.51		
Total	28	58.06				28	2652.90			

Significant codes: 0 “∗∗∗”; 0.01 “∗∗”; 0.05 “∗”.

**Table 4:** Optimized responses and theoretical optimum.

Dependent variable	*X*_1_	*X*_2_ (min)	*X*_3_ (mL/g)	Optimal value^†^	Theoretical predicted^‡^
Pectin yield (%)	2.00	62.07	36.57	24.32	24.30
DE (%)	2.59	70.05	40.00	78.26	73.00
MeO (%)	2.23	60.97	31.12	10.72	10.45
AUA (%)	2.15	57.77	26.97	83.24	81.33

^†^Optimum calculated by each model separately. ^‡^Responses at pH = 2.0, extraction time = 62.31 min, and LSR = 35.07 g/mL.

**Table 5:** Chemical properties of pectin obtained from watermelon rind compared with standard pectin.

Properties	Standard pectin	Extracted pectin
Ash (%)	3.77 ± 3.39	1.24
Alkalinity of ash (calcium carbonate,%)	2.34 ± 2.90	0.39
Free acid (meq/g)	0.78 ± 0.46	1.25
MeO (%)	≥6.70 (USP)	10.66
DE (%)	≥50 high methoxyl pectin 71-74 rapid set	73.33
AUA (%)	≥74 (USP) ≥65 (FAO/WHO)	82.50

## Data Availability

The experimental results of pectin extraction obtained from BBD design, R-Program for ANOVA analysis, and IR-figure of pectin data used to support the findings of this study have been deposited in the Watermelon Pectin Mendeley repository (Perez, Jose [59], “Watermelon Pectin”, Mendeley Data, V2, doi:10.17632/fhnyv7zspj.2). Correspondence and requests for materials should be addressed to Jose Perez.
